# Aroma of *Genius* Essential Oil Blend Significantly Enhances Cognitive Performance and Brain Metabolism in Healthy Adults

**DOI:** 10.1002/hup.70027

**Published:** 2025-11-19

**Authors:** Mark Moss, Jake Howarth, Holly Moss

**Affiliations:** ^1^ Department of Psychology, Faculty of Health and Wellbeing Brain Performance and Nutrition Research Centre Northumbria University Newcastle Upon Tyne UK

## Abstract

This double‐blind positive‐controlled study investigated the potential for the aroma of a novel blend of essential oils, *Genius* to enhance cognitive performance and mood in healthy adults, and whether any such benefits might be related to changes in cerebrovascular oxygenation measured using Near Infra‐Red Spectroscopy. Ninety participants (61 female) were pseudo‐randomly allocated to achieve a gender balance across three experimental groups: *Genius* aroma, Sage aroma (positive control) or no aroma (control). All participants completed mood questionnaires after completing a range of cognitive tasks whilst wearing a Near Infra‐Red Spectroscopy headband. Multivariate and subsequent univariate data analysis revealed significant enhancements to memory and executive function tasks in the *Genius* and sage aroma conditions compared to no aroma with larger effects noted for the *Genius* blend. Furthermore, the novel blend outperformed the aroma of pure sage and also left participants feeling significantly more alert and less fatigued at the end of the testing session. Near Infra‐Red Spectroscopy data indicated that both sage and *Genius* blend enhanced metabolism during task performance with a greater impact from the *Genius* aroma. Although suggestive of a mechanism underpinning the enhancements observed no correlations were found between the Near Infra‐Red Spectroscopy signals and cognitive performance. This study strengthens the evidence base for the beneficial effects of essential oil aroma inhalation for cognitive performance, however the underlying mechanisms remain elusive.

## 1 Introduction

The potential for inhaled aromas of essential oils and blends to impact positively on human behaviour and subjective state is an area of increasing interest in complimentary medicine (Vora et al. 2024), healthy ageing (Chavda et al. 2025) and general wellbeing (Caballero‐Gallardo et al. 2025). One area that has received particular attention is that of cognitive performance. Early research by Ludvigson and Rottman (1989) demonstrated that lavender aroma impaired arithmetic reasoning but not word recall task performance. Interestingly this effect was not present in a second test session a week later. The authors suggest that these effects might be explained by complex relationships between individual differences and relaxation as outlined by Lawless (1991) although they claim no direct support of this from their data. In contrast, Diego et al. (1998) reported that both lavender and rosemary positively affected different dimensions of mood. Lavender delivered overall better mood as measured by the profile of mood states questionnaire and rosemary increased alertness. Both aromas reduced anxiety and increased feelings of relaxation whilst also improving the speed of simple arithmetic computations. Although both aromas also led to an increase in accuracy on this measure only the effect for lavender reached statistical significance. Electroencephalogram (EEG) band activity data gathered at the same time supported the hypotheses that lavender was relaxing and rosemary alerting. Diego et al. (1998) suggest that both increased alertness and relaxation may be beneficial for the maths computations by facilitating concentration on the task but recognised that further research was required on the underlying mechanisms of the observed effects.

Computerised cognitive assessment has permitted more detailed investigation into the effects of aromas of single essential oils on a range of cognitive processes (M. Moss et al. 2003, 2008). The reported data suggest that tasks with a higher memory load such as word recall are more sensitive to the enhancing effects of aroma inhalation than are attentional tasks such as simple and choice reaction time. The computerised tasks employed in the current study match those previously employed for investigations of sage essential oil (D. O. Kennedy et al. 2006; L. Moss et al. 2010; Scholey et al. 2008; N. T. Tildesley et al. 2003). The findings of these studies identified enhancements for aspects of memory and mental arithmetic, leading to the choice of sage as a suitable positive control here. The majority of published research into the effects of aromas of essential oils has been based on single extract commercially available oils for example (Afghan et al. 2024; Filiptsova et al. 2017; Ito and Kawahara 2018; Sazawa et al. 2022). However, the synergistic blending of essential oils is at the heart of aromatherapy (Rhind 2015) although the actual efficacy of blending has received limited attention, and studies of blends that have reported significant benefits often do not include positive controls for example (M. Moss et al. 2025). One study that made a direct comparison of pure lavender oil to a commercial blend found nothing to recommend the blend above the single oil in terms of anxiety buffering effects (Swinburne et al. 2023). The producers of *Genius* created the blend based on two principles; evidence of psychological benefits for the component oils, coupled with aromatic hedonics of the final blend. Specifically, Patchouli has been shown to partially reverse cognitive impairments in animal models (Lin et al. 2024; Xu et al. 2022). Cardamom contains around 30% 1,8‐cineole (Ashokkumar et al. 2020), a compound that has previously been linked to cognitive enhancement in healthy adults (M. Moss and Oliver 2012). Frankincense (Khajehdehi et al. 2022) and Lemongrass have also been shown to improve attention and memory and subjective alertness (Verma 2025). Grapefruit increases sympathetic activity (Haze et al. 2002) whilst Neroli is an anxiolytic and analgesic (Scandurra et al. 2022) and Spikenard possesses sedative properties (Takemoto et al. 2015). It is possible that the blend of oils may produce synergistic effects whereby the whole is greater than the sum of its parts, a precedent for which exists in analgesia (Chabot‐Doré et al. 2015) and anaesthetics (Hendrickx et al. 2008) and a theoretical perspective that underpins essential oil therapy (Harris 2002).

Four potential mechanisms have previously been proposed to underpin the effects of essential oil inhalation on human behaviour. Hedonic valence outlines how our evaluation of the pleasantness of an aroma might be a predictor of the influence that aromas have on our behaviour (Broughan 2002). A quasi‐pharmacological mechanism is based on the observation that the olfactory bulb has direct connections to many areas of the brain including the amygdala‐hippocampal complex. The amygdala is important for the experience of emotion (Šimić et al. 2021) and the hippocampus has long been identified as critical for learning and memory, see Bird and Burgess (2008) for a review. The potential for essential oils to also exert a pharmacological mechanism has also been explored. Active components can be absorbed via respiration, cross the blood brain barrier, and thus produce systemic effects (Agatonovic‐Kustrin et al. 2019). It has also been argued that the effects of the aromas of essential oils are likely to be influenced by expectancy bias (Howard and Hughes 2008). A number of studies have investigated such a possibility, for example Köteles and Babulka (2014) reported that participants' expectations predicted changes in alertness in the case of rosemary and lavender oils but had no impact on associated cardiovascular variables.

A possible mechanism that has been largely overlooked in relation to aromatherapy is through an impact on brain metabolism. Previous research into chronic herbal supplementation has demonstrated potential for enhancement of cognition and brain activation as measured using functional magnetic resonance imaging (fMRI) (Carmichael et al. 2018; Zhang et al. 2014). Acute beneficial effects have also been reported using the functional near infra‐red spectroscopy (fNIRS) analogue (Best et al. 2021; M. Moss et al. 2018). Although fMRI has been employed in aroma research this has been limited to investigations of functional connectivity during aroma exposure (Martial et al. 2023), odour memory (Levy et al. 1999), and food‐related odours and reward (Frasnelli et al. 2015). A systematic review of fNIRS in olfactory research identified a clear focus on cortical activation in response to different odours across genders and population types (Gunasekara et al. 2022). One study has demonstrated a positive impact of phytoncide inhalation on Stroop task performance and haemodynamic response measured using fNIRS employing an elderly sample with mild cognitive impairment (Park et al. 2024). However, the current study is the first that the authors are aware of that focuses specifically on assessing the potential for a novel essential oil blend to deliver cognitive enhancement and impact on brain metabolism in health young adults.

We aimed to further knowledge by comparing a commercial blend created to impact on cognition to a positive control, a single essential oil aroma previously reported to have positive effects, and a no aroma control condition.

Based on the evidence summarised above we hypothesised:

1. The aromas of sage essential oil and *Genius* essential oil blend will enhance cognition particularly for memory based tasks compared to a no aroma control condition.
2. Both aromas will positively impact on mood compared to the control condition.
3. Both aromas will impact positively on brain metabolism measured using fNIRS.

A major question to be answered by this study was whether the essential oil blend would lead to greater enhancement of cognition than the single oil. It was not possible to specify this a priori.

## 2 Method

### 2.1 Design

A double‐blind one factor independent groups design was employed. The independent variable was the aroma condition and had three levels: *Genius* aroma, sage aroma (positive control) or no aroma (control). The dependent variables were the scores on the cognitive tasks, self‐reported mood and Near Infra‐Red Spectroscopy measures of oxygenated, deoxygenated and total haemoglobin. Research staff were blind to the aroma condition they were applying, and participants were not informed of the inclusion of aroma until the debrief. Any participants who commented on the presence of the aroma in the test room were told that it was left over from a previous user and not part of the current study. This was done to try and counter any possible expectancy effects.

### 2.2 Sample Size Calculation

G*Power was used to calculate the required sample size for a MANOVA main effect with three groups and 12 cognitive performance outcome variables. Parameters were set at *α* = 0.05, power of 0.8 and effect size *f* ^2^ = 0.3. This produced an indicative sample size of 90 participants. An approach of a global MANOVA test for significance followed by evaluation of effect sizes for individual dependent variables was adopted, with nominal levels of significance presented for information only. This follows the American Psychological Association guidelines for examining effects using small sample sizes (Wilkinson 1999).

### 2.3 Participants

Ninety healthy adults were each paid £10 for taking part in the study. All participants completed a standard health screen questionnaire and none were excluded on the basis of their responses. Previous use of essential oils was not recorded or controlled for. Participants were randomly allocated to the conditions as follows. *Genius* aroma, 12 males *M* = 19.75 years, SD = 1.87 and 18 females *M* = 20.22 years, SD = 2.65. Sage aroma, 14 males *M* = 20.07, SD = 1.33 and 16 females *M* = 20.44 years, SD = 2.58. Control 13 males *M* = 20.59, SD = 2.43 and 17 females *M* = 19.76 years, SD = 2.22. The three conditions did not differ significantly in age F(2, 84) = 0.972, *p* = 0.382 or in the number of males and females χ^2^(2) = 0.271, *p* = 0.873.

### 2.4 Materials

#### 2.4.1 Testing Cubicle

The testing cubicle measured 2.4 m long × 1.8 m wide × 2.4 m high and was maintained at a temperature between 18 and 22 degrees Celsius throughout the testing sessions.

#### 2.4.2 Aromas

*Genius* is a proprietary blend that includes Patchouli, Neroli, Grapefruit, Cardamom, Frankincense, Spikenard, Rosemary and Lemongrass essential oils. It has been created by Moods UK who maintain knowledge and ownership of the exact constituent components.

Sage essential oil aroma was employed as a positive control following previous demonstrations of its impact on cognition and mood in healthy adults employing similar tasks to those used here. N. Tildesley et al. (2005) reported significant improvements for oral sage treatment on serial subtractions, word recall and alert, calm and content mood scores in healthy adults. The improvements in word recall memory were confirmed by Scholey et al. (2008) in a sample of healthy older volunteers. L. Moss et al. (2010) further reported the benefits of sage aroma on word recall and picture recognition alongside an increase in alertness in healthy young adults.

Eight drops of the appropriate oil (or water in the control condition) were applied to a diffuser pad for a ‘Tisserand Aroma‐stream’. The Aroma‐stream was placed under the bench in the testing cubicle so as to be out of sight, and switched on for 15 min prior to the testing of each participant. Each aroma was above detection threshold and of approximately equivalent strength for each testing session as assessed by an independent party. This was established prior to the start of the data collection by the lead author who set up the test cubicle on different days with varying amounts of oils and duration of dispersion. The independent party then rated the cubicle aroma on rating scales of strength and intensity. This led to the final diffusion protocol.

#### 2.4.3 Cognitive Tasks

All cognitive and mood measures were delivered using the Computerised Mental Performance Assessment System (COMPASS, Northumbria University, Newcastle upon Tyne, UK). COMPASS has been used in a number of herbal and nutritional intervention studies (Dodd et al. 2020; D. Kennedy et al. 2018; Wightman et al. 2021). The tasks were chosen to target aspects of memory and complex information processing that previous research has indicated may be sensitive to aromas (M. Moss et al. 2003; M. Moss and Oliver 2012). The battery lasted approximately 25 min and was constituted of the following:

##### 2.4.3.1 Word Presentation

Fifteen words are presented on the screen, one at a time, at the rate of 1 per second.

##### 2.4.3.2 Immediate Word Recall

Participants are given 60 seconds to orally recall as many words as possible from the list presented.

##### 2.4.3.3 Corsi Blocks

Nine blue squares are presented on a black background. Some of the squares change to red and then back to blue again in a sequence. Participants are required to remember the sequence and are required to use the mouse to click the blocks in the exact sequence in which they changed back to blue. The task is repeated three times at each level of difficulty, starting with four blocks in the sequence, until the participant can no longer correctly recall the sequences. The task continues up to a maximum nine‐block sequence, if participants are still making correct responses. The task was scored as an average of the last 3 correctly completed trials; for example, if the participant responds correctly to all three Level 5 trials and only one Level 6 trial their span score would be 5.3 [(5 + 5 + 6)/3].

##### 2.4.3.4 Numeric Working Memory

Five digits between 1 and 9 are displayed on the screen, one at a time at a rate of 1 per second. Participants are required to memorise the numbers as they appear. Once the series is complete, numbers are displayed one at a time and the participants are required to indicate if each of the numbers were presented in the original list or not. Participants respond using buttons for ‘Yes’ and ‘No’ on a response pad. Three repetitions are presented during the task. Participants are scored on their overall accuracy and reaction time (msec) for these responses.

##### 2.4.3.5 Serial Subtraction of Threes and Sevens

A random number (between 800 and 999) is presented on the screen. Participants are required to subtract a number (either 3 or 7) from the starting number and continue to do this for the duration of the task. Only the starting number is shown on the screen and the rest of the numbers are generated from the participant's previous answers. Participants use the keyboard to type their responses and press enter after each response. Each task lasts 2 min in total, with serial 7s being completed immediately after serial 3s. Number of correct responses and errors are recorded.

##### 2.4.3.6 Rapid Visual Information Processing (RVIP)

A series of single digit numbers are presented on the screen at a rate of 100 per minute. The participant is required to monitor this and respond by pressing the centre button on the response pad as quickly as possible when they saw 3 odd or 3 even numbers in a row. The task lasts 5 min in total, with 8 correct target strings presented each minute. The task is scored for percentage of target strings correctly detected and mean reaction time for correct detection (msec).

##### 2.4.3.7 Delayed Word Recall

Participants are given 60 seconds to orally recall as many words as possible from the original list presented at the start of the cognitive assessment.

##### 2.4.3.8 Alertness and Mental Fatigue Visual Analogue Scales (VAS)

The scales showed a 100 mm line with ‘Not at all’ and ‘Extremely’ at either end on which participants mark their current subjective evaluation.

#### 2.4.4 Functional Near InfraRed Spectroscopy (NIRS)

Functional NIRS is a brain‐imaging technique that is founded on the inherent optical absorption properties of oxygenated and deoxygenated haemoglobin when near‐infrared light is projected through the skull. When assessed by NIRS, an increase in cerebral blood flow in the cortex is seen as an increase in the total concentration of haemoglobin and a concomitant decrease in deoxy‐haemoglobin (Steinbrink et al. 2006). Relative changes in the absorption of near infrared light were measured at a time resolution of 10 Hz by using a 12‐channel Oxymon system (Artinis Medical Systems BV, Zetten, Netherlands). The system emitted 2 nominal wavelengths of light (765 and 855 nm) with an emitter/optode separation distance of 4 cm. All NIRS output data were time stamped at the beginning of each task to ensure that data corresponded to the relevant epoch of task performance as previously reported (M. Moss et al. 2018).

### 2.5 Procedure

The study and all its procedures were risk assessed and approved by the Faculty of Health and Life Sciences ethics committee prior to any data collection, record #8212. All testing took place between 9:00 and 12:00 to reduce any time of day effects, and participants were requested to eat a light breakfast and refrain from caffeine consumption for 1 hour prior to testing. Participants were tested individually and randomly allocated to aroma condition prior to attending the laboratory. Participants were seated in the test cubicle that was infused with aroma based on assigned condition. They were then fitted with the NIRS headband and after a 5‐min baseline period the cognitive assessments were completed. The cognitive assessment NIRS data were split into epochs of individual task performance; The length of the Corsi blocks task depended on participant's individual performance, therefore NIRS data from just the first 3 minutes of the task was analysed—as this was the minimum length of time the task would last. Following completion of the cognitive assessment, the NIRS equipment was removed and the participants completed the questionnaires before being fully debriefed. The whole testing visit lasted about 45 min in total.

## 3 Results

### 3.1 Cognitive Performance

Due to the number of dependent variables in this study, an initial MANOVA was conducted to establish if the aroma treatments differed along a combination of all these variables, whilst protecting against inflated type 1 error due to multiple tests of correlated dependent variables (Tabachnick and Fidell 2007). Box's test of homogeneity of covariance matrices produced a significant result *M* = 298.812, F(156, 20167.943) = 1.536, *p* < 0.001. However, with equal group sizes greater than 30, MANOVA tends to be robust to violations with the Wilks' lambda statistic performing well in conditions of heterogeneity of covariance matrices (Ateş et al. 2019). The MANOVA revealed a significant effect of condition on the combined variables Wilks' lambda = 0.620, F(24, 152) = 1.707, *p* = 0.029, partial eta squared = 0.212. Levene's tests indicated that only the serial threes variables presented significant heterogeneity of variance. A correction for multiple testing removing these significant effects. Follow up univariate ANOVAs identified a number of variables for which medium sized effects existed for the difference between conditions. These are presented in Table 1 along with the uncorrected omnibus significance values for information only, and Tukey pairwise comparisons. As can be seen, the *Genius* aroma produced significantly better performance than the no aroma control condition for seven of the cognitive performance measures, specifically those related to memory and complex processing.

**TABLE 1:** Mean(SD) values for cognitive performance tasks for each condition.

Variable	Sage (positive control)	*Genius*	Control	Uncorrected sig	Partial eta squared	Significant comparisons	Cohen's *d* [95% CI] for comparisons
NWM correct (%)	95.7 (5.3)	96.4 (5.0)	92.0 (8.8)	0.024	0.082	*Genius* versus Control*	0.619 [0.098, 1.135]
NWM speed (msec)	1035.3 (212.9)	908.1 (261.2)	1024.4 (205.9)	0.062	0.062		
Corsi block span	6.0 (0.9)	6.3 (0.9)	5.7 (0.7)	0.051	0.066	*Genius* versus Control*	0.671 [0.148, 1.189]
Serial 3s correct	25.6 (12.2)	31.3 (10.5)	22.7 (7.8)	0.006	0.110	*Genius* versus Control**	0.927 [0.391, 1.457]
Serial 3s errors	2.0 (1.9)	2.4 (2.3)	3.6 (3.2)	0.040	0.071	Sage versus Control*	0.614 [0.093, 1.130]
Serial 7s correct	16.2 (8.8)	19.2 (9.4)	13.6 (6.9)	0.038	0.072	*Genius* versus Control*	0.686 [0.162, 1.204]
Serial 7s errors	2.5 (2.8)	2.7 (2.1)	5.6 (9.2)	0.068	0.060		
RVIP correct (%)	48.0 (22.4)	55.4 (20.5)	41.1 (17.3)	0.027	0.080	*Genius* versus Control*	0.754 [0.227, 1.276]
RVIP speed (msec)	479.6 (53.6)	464.9 (48.0)	490.7 (66.8)	0.217	0.035		
RVIP false alarms	7.2 (7.6)	6.0 (5.9)	8.7 (7.2)	0.323	0.026		
IWR correct	6.6 (1.4)	7.9 (1.4)	6.5 (1.9)	0.002	0.138	*Genius* versus Control** *Genius* versus Sage**	0.836 [0.304, 1.361] 0.894 [0.359, 1.422]
DWR correct	4.9 (2.0)	6.0 (2.0)	4.6 (1.8)	0.024	0.082	*Genius* versus Control*	0.698 [0.173, 1.216]

*Note:* Correct scores indicate total number of correct responses except where indicated as percentages. Reaction times are in milliseconds. Uncorrected sig values are drawn from the univariate ANOVAs. Significant comparisons are Tukey pairwise comparisons from significant ANOVAs.

Abbreviations: DWR, delayed word recall; IWR, immediate word recall; NWM, numeric working memory; RVIP, rapid visual information processing.

^*^ *p* < 0.05.

^**^ *p* < 0.01.

### 3.2 Mood

A similar approach was taken to the analysis of the two mood variables. Box's test indicated equality of covariance matrices, *M* = 7.241, F(6, 1888642.769) = 1.167, *p* = 0.321. The MANOVA revealed a significant difference between groups on the combined mood variables, Wilks' lambda = 0.741, F(4, 172) = 6.948, *p* < 0.001, partial eta squared = 0.139. Levene's test indicted homogeneity of variance for both Alert and Fatigue variables. Univariate ANOVAs show significant differences exist between conditions for participants' ratings of both Alert and Fatigue at the end of the testing procedure. Descriptive statistics, uncorrected significance levels, effect sizes and Tukey comparisons are presented in Table 2.

**TABLE 2:** Mean (SD) values for the subjective mood measures.

Variable	Sage (positive control)	*Genius*	Control	Uncorrected sig	Partial eta squared	Significant comparisons	Cohen's *d* [95% CI] for comparisons
Alert	48.5 (16.8)	57.4 (16.9)	38.6 (13.0)	< 0.001	0.200	*Genius* versus Control** Sage versus Control*	1.251 [0.691, 1.801] 0.661 [0.138, 1.178]
Fatigue	48.6 (20.8)	36.4 (19.1)	55.0 (19.2)	0.002	0.136	*Genius* versus Control** *Genius* versus Sage*	−0.967 [−1.499, −0.428] −0.609 [−1.124, −0.088]

*Note:* Uncorrected sig values are drawn from the univariate ANOVAs. Significant comparisons are Tukey pairwise comparisons from significant ANOVAs. Pearson correlations revealed no significant relationships between mood and performance save for between fatigue and immediate word recall r(88) = −0.216, *p* = 0.041, a small effect.

^*^ *p* < 0.05.

^**^ *p* < 0.01.

### 3.3 NIRS

The NIRS data total haemoglobin, oxygenated haemoglobin and deoxyhaemoglobin were analysed using mixed (condition × task epoch) ANOVA with the multivariate approach favoured for reporting the repeated measures (task epoch) factor due to a significant violation of the assumption of sphericity.

### 3.4 Total Haemoglobin

**FIGURE 1:**
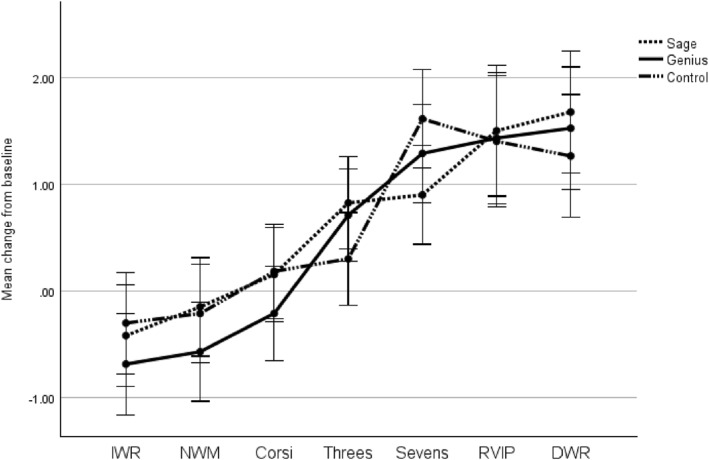
Mean change from baseline in concentration of total haemoglobin during cognitive assessment. Error bars represent standard errors. DWR, Delayed word recall; IWR, Immediate word recall; NWM, Numerical working memory; RVIP, Rapid visual information processing.

No significant main effect of condition was found F(2, 87) = 0.027, *p* = 0.973, partial eta squared = 0.001. A significant main effect of task epoch was present, Wilks' lambda = 0.426, F(6, 82) = 18.423, *p* < 0.001, partial eta squared = 0.574. Total haemoglobin levels increased steadily over the cognitive testing period. No significant condition*task epoch interaction effect was present, Wilks' lambda = 0.808, F(12, 164) = 1.538, *p* = 0.115, partial eta squared = 0.101. (See Figure 1).

### 3.5 Oxygenated Haemoglobin

**FIGURE 2:**
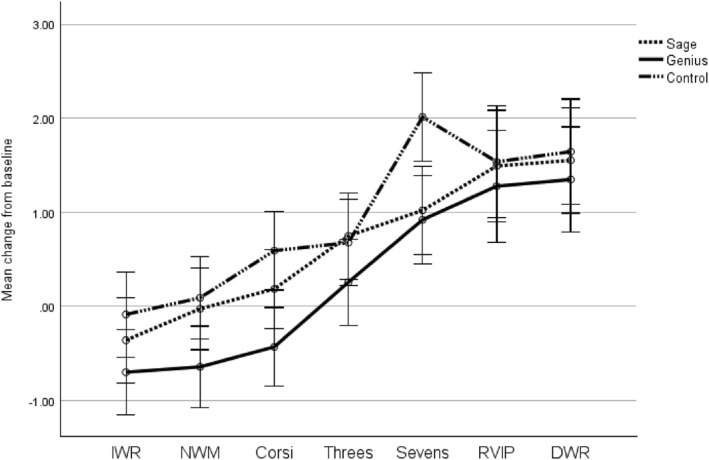
Mean change from baseline in concentration of Oxyhaemoglobin during cognitive assessment. Error bars represent standard errors. DWR, Delayed word recall; IWR, Immediate word recall; NWM, Numerical working memory; RVIP, Rapid visual information processing.

No significant main effect of condition was found F(2, 87) = 0.499, *p* = 0.609, partial eta squared = 0.011. A significant main effect of task epoch was present, Wilks' lambda = 0.383, F(6, 82) = 22.020, *p* < 0.001, partial eta squared = 0.617. In addition, a significant condition*task epoch interaction effect was present, Wilks' lambda = 0.764, F(12, 164) = 1.964, *p* = 0.031, partial eta squared = 0.126 (see Figure 2).

### 3.6 Deoxygenated Haemoglobin

**FIGURE 3:**
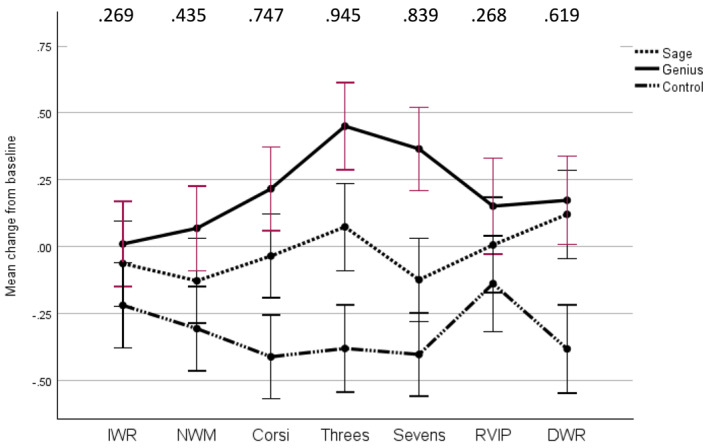
Mean change from baseline in concentration of Deoxyhaemoglobin during cognitive assessment. Error bars represent standard errors. Values at the top of the figure are the Cohen's d values for comparisons between the *Genius* and Control conditions. DWR, Delayed word recall. IWR, Immediate word recall; NWM, Numerical working memory; RVIP, Rapid visual information processing.

A significant main effect of condition was identified F(2, 87) = 4.659, *p* = 0.012, partial eta squared = 0.097, a medium effect. No significant main effect of task epoch was present, Wilks' lambda = 0.924, F(6, 82) = 1.126, *p* = 0.354, partial eta squared = 0.076 and no Condition*task epoch was found, Wilks' lambda = 0.836, F(12, 164) = 1.283, *p* = 0.233, partial eta squared = 0.086 (see Figure 3).

### 3.7 Correlations

Pearson correlations were performed between the cognitive performance measures, NIRS signals and mood variables. See Table 3. To summarise, based on Cohen's criteria (Cohen 1992) there is just one medium sized effect that between Deoxyhaemoglobin levels and serial threes correct. Thirty‐three are small effects and the rest are negligible.

**TABLE 3:** Pearson correlations between cognitive performance measures, NIRS signals and mood variables, *N* = 90.

	Total Hb	Oxy Hb	Deoxy Hb	Alert	Fatigue
NWM correct (%)	−0.065	0.034	−0.001	0.093	−0.147
NWM speed (msec)	0.029	0.044	−0.009	−0.092	−0.070
Corsi block span	−0.074	−0.154	0.199	0.167	−0.123
Serial 3s correct	−0.093	−0.209*	0.317*	0.117	−0.035
Serial 3s errors	0.186	0.213*	−0.097	−0.097	0.154
Serial 7s correct	−0.221*	−0.196	−0.053	0.110	−0.043
Serial 7s errors	0.100	0.121	−0.070	0.020	0.043
RVIP correct (%)	−0.067	−0.079	0.031	0.096	−0.123
RVIP speed (msec)	0.121	−0.076	−0.161	0.021	−0.003
RVIP false alarms	0.203	0.229*	−0.065	−0.040	0.141
IWR correct	−0.048	−0.122	0.204	0.203	0.216*
DWR correct	−0.052	−0.086	0.106	0.077	−0.089

Abbreviations: DWR, Delayed word recall; IWR, Immediate word recall; NWM, Numerical working memory; RVIP, Rapid visual information processing.

^*^ *p* < 0.05 (uncorrected).

## 4 Discussion

The data reported here indicate that the inhalation of the aromas of essential oils can significantly improve cognitive performance on a range of tasks. Furthermore, that the *Genius* blend of essential oils developed specifically for the potential to impact on cognition does so to a greater extent than the aroma of sage essential oil alone that has previously been demonstrated to have beneficial effects (L. Moss et al. 2010; M. Moss et al. 2014). Furthermore, this study is the first to employ Near Infra‐Red Spectroscopy (NIRS) to measure brain metabolism during cognitive task performance under conditions of aroma inhalation. The NIRS data clearly indicate differences in oxygen utilisation during completion of the tasks which appears consistent with greater metabolism being associated with better performance (Dienel 2018).

Synergistic effects of essential oils and essential oil components have previously been established in the fields of antibiotics (Langeveld et al. 2014), anticancer (Lesgards et al. 2014) and food preservation (Hyldgaard et al. 2012) and have long been proposed as underpinning aromatherapy (Harris 2002). However, this is the first time the authors are aware of where potential synergistic effects have been demonstrated for the impact of essential oil aromas on cognitive performance. The beneficial effects on memory of the individual components, Patchouli, Frankincense and Cardamom that constitute the *Genius* blend are typically small to medium sized for example (Asadi et al. 2019; Beheshti et al. 2020; Setty et al. 2013; Stafford et al. 2009) although some of the studies employ animal models and oral rather than aroma based interventions which makes direct comparison to the current study difficult. Human studies of grapefruit aroma demonstrate enhanced vigilance evidenced by P300 evoked potentials (Ohno et al. 2007) and physiological arousal, blood pressure and heart rate (Kawai et al. 2020) although these were not reflected in cognitive data. In contrast, studies of lemongrass aroma in isolation have shown medium sized cognitive enhancements (Shrimali et al. 2024) including for tasks of the kind employed in the current study (Verma 2025). Importantly however, the data reported here indicate medium to large beneficial effects for the *Genius* blend when compared to the no aroma control based on Cohen's criteria (Cohen 1992). Whilst not being in any way definitive, these findings offer support for the potential for synergy, with the effects of combined oils being greater than the individual oils alone. Research employing fMRI has confirmed that blended aromatic components naturally fuse to form a unique odour that is qualitatively different from each component and favours configural processing in the inferior frontal gyrus (Sinding et al. 2021). Such unique processing of blended components may be responsible at least in part for synergistic effects on behaviour.

When considering the single essential oil sage positive control, the size of the effects on cognitive performance observed here are similar to those previously reported for both the aroma (L. Moss et al. 2010) and oral administration of the oil (Scholey et al. 2008). The work of Scholey and colleagues also demonstrated that the sage extract possesses anticholinesterase properties and argue that this may account for the cognition enhancing properties. Certainly, it cannot be solely due to aromatic properties as the extract was taken in a double‐blind capsule form in their study. Detectable serum levels of compounds with similar enzyme inhibiting properties have previously been found following inhalation of the aroma of rosemary essential oil and these correlated with performance on some of the tasks employed in the current study (M. Moss and Oliver 2012). It is possible that a similar pharmacological mechanism underpins some of the effects observed here although no serum analysis was performed and so this cannot be confirmed. An alternative, or possibly parallel mechanism may the through the direct stimulation of the brain by the olfactory bulb during aroma exposure. The olfactory system is the most direct of our senses (Fontanini and Katz 2008), with olfactory stimulation passing just one synapse to reach the olfactory bulb (Li et al. 2011). The olfactory bulb then processes the olfactory inputs across a series of laminar structures before distributing the processed information to the olfactory cortices, many of which support cognition such as the orbitofrontal cortex, amygdala, pyriform cortex, and entorhinal cortex (Leon and Woo 2018). It is possible that in part at least this stimulation during cognitive task completion is responsible for improved performance. Interestingly, loss of olfaction is associated with volume loss in the same areas of the brain (Yao et al. 2014) further linking olfactory stimulation to cognitive performance.

Cognitive task performance increases aerobic glucose metabolism in the brain (Al‐Naher et al. 2016), and supplementation of neural substrates have previously been demonstrated to enhance cognitive performance (M. C. Moss and Scholey 1996; Scholey et al. 2001). The NIRS measures of total blood flow and oxygenated haemoglobin in the current study show clear increases over the period of cognitive task performance in all conditions, something that represents the demand for ‘fuel’. Such effects on the NIRS signals have been previously demonstrated in response to cognitive effort (Witte et al. 2015). What is of particular interest here is that levels of deoxygenated haemoglobin were at significantly higher levels during cognitive task completion in the *Genius* aroma condition and to a lesser extent in the sage aroma condition compared to the no aroma control condition. We argue that this pattern of results indicates oxygen extraction may be increased in the aroma conditions in response to cognitive demand. Something that is less available in the control condition. The argument that links such NIRS signals to cognition has been made previously (Bönöczk et al. 2002; D. O. Kennedy et al. 2010), although Kennedy et al. reported no impact on cognition associated with such an increase. In the current study a relatively consistent increase in deoxygenated Haemoglobin was observed across the testing period but not all tasks benefited in terms of enhancement by the *Genius* or sage aromas. If increased oxygen extraction and associated glucose metabolism are the key to cognitive enhancement then perhaps all measures might be expected to have been affected. This introduces an interesting question as to whether the observed changes in cerebral blood flow are in fact related to cognitive performance, or simply artefacts of the aroma processing that do not relate to cognition? The correlational analyses suggest that changes in cerebral blood flow do not underpin the changes in cognition. Nearly all the correlations are small or very small based on Cohen's criteria (Cohen 1992) and none would even approach significance if a correction were made for multiple comparisons. Equally, the mood variables are not related to cognitive performance with the majority of the correlations being near zero which dismisses any suggestion of changes in cognition being a consequence of improved subjective state.

This evaluation leaves the most plausible potential mechanisms to be pharmacological or quasi‐pharmacological as outlined above. Neither of these can be confirmed or dismissed based on the data presented here. The employment of more structurally accurate brain imaging techniques such as fMRI would enable evaluation of metabolic changes that may be linked to changes in performance under conditions of aroma inhalation. Structural and functional alterations have previously been reported in pro‐gamers and these were linked to task appropriate improvements in performance (Choi et al. 2021). Although structural changes would not be expected during acute aroma exposure, metabolic changes in brain regions linked to task performance may be illuminating. Equally, bench studies of the pharmacological properties of *Genius* essential oil blend coupled with serum analysis of absorbed compounds could provide evidence of a pharmacological mechanism underpinning the effects observed here.

To conclude, the current study adds to the growing body of evidence indicating that cognitive functioning can be enhanced through the acute inhalation of the aromas of essential oils and their blends, and that such effects are not the consequence of generally increased pre‐frontal metabolism or changes in subjective mood state. This area requires further investigation to determine exactly how aromas facilitate such changes.

## Funding

This study was funded by Mood UK Ltd.

## Conflicts of Interest

The authors declare no conflicts of interest.

## Supporting information


Supporting Information S1

## Data Availability

The data that support the findings of this study are openly available in OSF at https://osf.io/dashboard, reference number https://doi.org/10.17605/OSF.IO/T6EMK.
