# Supplementary material for: Aroma of Genius Essential Oil Blend Significantly Enhances Cognitive Performance and Brain Metabolism in Healthy Adults
Source: Hum Psychopharmacol. 2025 Nov 19;40(6):e70027. doi: 10.1002/hup.70027 (PMC12628357; doi:10.1002/hup.70027)
Supplement: Supplementary file 1 — Supporting Information S1 [file HUP-40-e70027-s001.docx]

Supplementary Tables

Deoxyhaemoglobin

|  | Condition | Mean | Std. Deviation |
| --- | --- | --- | --- |
| Deoxy_IWR | Sage | -.0627 | .91074 |
|  | Genius | .0103 | .84836 |
|  | Control | -.2190 | .85875 |
| Deoxy_NWM | Sage | -.1277 | .86649 |
|  | Genius | .0687 | .85883 |
|  | Control | -.3063 | .86696 |
| Deoxy_Corsi | Sage | -.0347 | .89046 |
|  | Genius | .2167 | .86245 |
|  | Control | -.4117 | .81878 |
| Deoxy_Threes | Sage | .0740 | .92111 |
|  | Genius | .4507 | .91552 |
|  | Control | -.3807 | .84281 |
| Deoxy_Sevens | Sage | -.1237 | .70201 |
|  | Genius | .3657 | .95194 |
|  | Control | -.4030 | .87796 |
| Deoxy_RVIP | Sage | .0067 | .73273 |
|  | Genius | .1517 | 1.19371 |
|  | Control | -.1380 | .95481 |
| Deoxy_DWR | Sage | .1210 | .91119 |
|  | Genius | .1737 | 1.05973 |
|  | Control | -.3823 | .69983 |

Oxyhaemoglobin

|  | Condition | Mean | Std. Deviation |
| --- | --- | --- | --- |
| Oxy_IWR | Sage | -.3583 | 2.29643 |
|  | Genius | -.6987 | 2.93846 |
|  | Control | -.0850 | 2.11749 |
| Oxy_NWM | Sage | -.0247 | 2.11745 |
|  | Genius | -.6417 | 2.87109 |
|  | Control | .0923 | 2.07436 |
| Oxy_Corsi | Sage | .1860 | 2.22857 |
|  | Genius | -.4297 | 2.59624 |
|  | Control | .5927 | 2.00760 |
| Oxy_Threes | Sage | .7490 | 2.36085 |
|  | Genius | .2577 | 2.85190 |
|  | Control | .6793 | 2.28869 |
| Oxy_Sevens | Sage | 1.0213 | 2.36797 |
|  | Genius | .9210 | 3.03220 |
|  | Control | 2.0137 | 2.27382 |
| Oxy_RVIP | Sage | 1.4930 | 2.50340 |
|  | Genius | 1.2783 | 4.42222 |
|  | Control | 1.5383 | 2.41438 |
| Oxy_DWR | Sage | 1.5537 | 2.60087 |
|  | Genius | 1.3493 | 3.81839 |
|  | Control | 1.6447 | 2.60683 |

Total Haemoglobin

|  | Condition | Mean | Std. Deviation |
| --- | --- | --- | --- |
| Total_IWR | Sage | -.4210 | 2.71832 |
|  | Genius | -.6883 | 2.95668 |
|  | Control | -.3040 | 2.06804 |
| Total_NWM | Sage | -.1523 | 2.46426 |
|  | Genius | -.5730 | 3.02434 |
|  | Control | -.2140 | 1.99700 |
| Total_Corsi | Sage | .1513 | 2.33840 |
|  | Genius | -.2130 | 2.78846 |
|  | Control | .1810 | 2.09232 |
| Total_Threes | Sage | .8230 | 2.34472 |
|  | Genius | .7083 | 2.48509 |
|  | Control | .2987 | 2.29995 |
| Total_Sevens | Sage | .8977 | 2.24535 |
|  | Genius | 1.2867 | 2.74849 |
|  | Control | 1.6107 | 2.57875 |
| Total_RVIP | Sage | 1.4997 | 2.38248 |
|  | Genius | 1.4300 | 4.67524 |
|  | Control | 1.4003 | 2.50868 |
| Total_DWR | Sage | 1.6747 | 2.42811 |
|  | Genius | 1.5230 | 4.05365 |
|  | Control | 1.2623 | 2.72436 |
